# Hierarchical Recursive Organization and the Free Energy Principle: From Biological Self-Organization to the Psychoanalytic Mind

**DOI:** 10.3389/fpsyg.2017.01695

**Published:** 2017-09-26

**Authors:** Patrick Connolly, Vasi van Deventer

**Affiliations:** ^1^Counselling and Psychology, Hong Kong Shue Yan University, Hong Kong, Hong Kong; ^2^Psychology, University of South Africa, Pretoria, South Africa

**Keywords:** psychoanalysis, neuropsychoanalysis, free energy principle, systems theory, hierarchical recursive organization

## Abstract

The present paper argues that a systems theory epistemology (and particularly the notion of hierarchical recursive organization) provides the critical theoretical context within which the significance of Friston's (2010a) Free Energy Principle (FEP) for both evolution and psychoanalysis is best understood. Within this perspective, the FEP occupies a particular level of the hierarchical organization of the organism, which is the level of biological self-organization. This form of biological self-organization is in turn understood as foundational and pervasive to the higher levels of organization of the human organism that are of interest to both neuroscience as well as psychoanalysis. Consequently, central psychoanalytic claims should be restated, in order to be located in their proper place within a hierarchical recursive organization of the (situated) organism. In light of the FEP the realization of the psychoanalytic mind by the brain should be seen in terms of the evolution of different levels of systematic organization where the concepts of psychoanalysis describe a level of hierarchical recursive organization superordinate to that of biological self-organization and the FEP. The implication of this formulation is that while “psychoanalytic” mental processes are fundamentally subject to the FEP, they nonetheless also add their own principles of process over and above that of the FEP. A model found in Grobbelaar (1989) offers a recursive bottom-up description of the self-organization of the psychoanalytic ego as dependent on the organization of language (and affect), which is itself founded upon the tendency toward autopoiesis (self-making) within the organism, which is in turn described as formally similar to the FEP. Meaningful consilience between Grobbelaar's model and the hierarchical recursive description available in Friston's (2010a) theory is described. The paper concludes that the valuable contribution of the FEP to psychoanalysis underscores the necessity of reengagement with the core concepts of psychoanalytic theory, and the usefulness that a systems theory epistemology—particularly hierarchical recursive description—can have for this goal.

## Introduction

A question that is at the heart of the neuropsychoanalytic project is the relationship between two levels of organization within the human organism, between the neurological level and the mental one. Professor Karl Friston's free energy principle (FEP henceforth) of biological self-organization has captured the imagination of many within both the neuroscientific and psychoanalytic fields as providing a very important new link in our understanding of the body-mind relationship. However, it is important to understand this development within some form of theoretical context that clarifies the correct implications that this development has within the growing science of neuropsychoanalysis, so that the importance of its role and influence is neither over- nor under-estimated.

It is the view of the present paper that a systems theory epistemology provides the critical theoretical context within which the significance of Friston's FEP is best understood. Systems theory provides the concept of a “recursive description” of organization of complex systems in the physical world, in which the FEP enters at a particular level of that hierarchical organization, which is the level of biological self-organization. This form of biological self-organization is in turn understood as foundational and pervasive to the higher levels of organization of the human organism that are of interest to both neuroscience as well as psychoanalysis.

The implication of adopting this hierarchical, recursive description of organization of the human organism is twofold. First, it implies that all levels of organization recursively superordinate to the level of biological self-organization (in this case the brain and the mind) must be constrained by the FEP. The second implication is that those recursively superordinate levels of the organism which are the brain and the mind, must also be subject to further principles of organization *not* fully explained by the FEP. Historically, the theoretical field of psychoanalysis reflected an effort to generate such superordinate principles of organization that obtain at the level of psychic organization. However, the field developed independently of neuroscience and biology, which meant that psychoanalytic theories were never adequately integrated within a hierarchical model of levels of organization of the human organism.

In this manner, it will be suggested that core constructs in psychoanalytic theory should be restated, in order to be located in their proper place within a hierarchical recursive organization of the (situated) organism. Further it is argued that a correct understanding of the place of the FEP in organizing the psyche in turn necessitates a restatement of the core Freudian concepts within a systems theory framework that can successfully integrate biological levels of organization with psychological ones.

This paper will first introduce systems theory and the notion of hierarchical recursive organization, and describe how findings in different sciences support such a notion of recursive levels of organization in nature. Next, the paper highlights a problem within the psychoanalytic literature, in which organizing principles such as the pleasure principle, have never been adequately connected to organizing principles in the nervous system or the body in general. Following this it is suggested that psychoanalytic principles of organization need to be restated within a recursive description of organization within the human organism which demonstrates its dependence on biological self-organization. The FEP is then described as a key regulatory principle of self-organization which recursively underlies psychoanalytic regulatory principles. The FEP is described here as a formalization of Maturana and Varela's (1980) concepts of autopoiesis (or self-making) and the structural coupling of the organism with its environment.

The importance of the FEP in bridging the physical material of the body and the nervous system with the level of organization of information and (Bayesian) beliefs—within the psychological domain—is clarified. This concept is then used to restate the question as to how the brain realizes the psychoanalytic mind as one of the evolution of different levels of systemic organization in which the concepts of psychoanalysis are viewed as describing a level of hierarchical recursive organization superordinate to that of biological self-organization and the FEP. The implication of this formulation is presented, which is that while “psychoanalytic” mental processes are fundamentally subject to the FEP, they nonetheless also add their own principles of process over and above that of the FEP.

The paper then presents an example of how psychoanalytic regulatory principles can be founded on biological self-organization through the model presented in Grobbelaar (1989) which offers a recursive bottom-up description of the organization of the “psychoanalytic” consciousness as dependent on the organization of language (and affect), which is itself founded upon the tendency toward autopoiesis within the organism. Meaningful consilience between Grobbelaar's model and the hierarchical recursive description available in Friston's (2010a) theory is described. The paper concludes that the valuable contribution of Friston's FEP to psychoanalysis underscores the necessity of reengagement with the core concepts of psychoanalytic theory, and the usefulness that a systems theory epistemology—particularly recursive description—can have for this goal.

## General systems theory and the self-organization of systems

When the field of general systems theory came to the fore toward the middle of the Twentieth century, the key purpose of this field was to offer an explanatory paradigm for how systems of various kinds came to regulate themselves, and generate their own principles of organization. While this question of self-regulation spanned a number of different fields, a very exemplary question was that of biological systems, and how biological systems appeared to regulate themselves, since their behavior is not directly regulated by their environment. This question can also be stated in terms of “bottom-up” or “top-down” forms of organization in a system, or how a system comes to develop top-down principles of self-regulation, that order the activity of lower-order levels of the system. The field of general systems theory was from the start also associated with the field of cybernetics, which is the science of regulation or control of systems, which emerged at roughly the same time as general systems theory (Wiener, 1965; Von Bertalanffy, 1969/2009).

The answer that began to emerge from general systems theory is that those self-regulatory or top-down principles of organization of systems emerged from the activity of the lower-order elements themselves (in other words from bottom-up activity). This principle was proposed as applying to a wide variety of phenomena, including inorganic ones such as complex weather patterns that emerge from the interactions of vast numbers of air and water molecules in the atmosphere (Wiener, 1965), or patterns of convection that emerge in heated liquids (Prigogine and Stengers, 1984).

This same principle was applied to the regulation of biological systems, including complex social behavior: an example of the emergence of a patterned hierarchy of social dominance among chickens, is found in Wiener and Schadé (1965) restated vividly here in Grobbelaar (1989):

“*… the pecking order [of chickens] which is generated through the interactions of the chickens is spontaneously generated out of the activity of pecking. So that the activity of pecking determines the pattern of dominance, which in turn determines who will peck who.” (p. 137)*.

Grobbelaar goes on to say that there are other factors that influence the pecking order, which is correct. However, the point being made is that the operation of the elements of a system and the interactions between them will generate a new form of organization which in turn comes to determine the activity of those constituent elements. We will see below that exactly the same circular causality emerged subsequently in physics; specifically in the context of synergetics where slow macroscopic (superordinate) modes of behavior enslave fast microscopic (subordinate) levels (Haken, 1983; Tschacher and Haken, 2007). In physics, this is known as the enslaving principle and emerges in things like the Center Manifold Theorem in dynamical systems theory (Carr, 1981).

What's important about this perspective is to note that though von Bertalanffy (together with others) sought to describe the self-regulation of systems based on bottom up processes, he also stressed the significance of aspects of holism and integration in the emergence of self-regulatory tendencies:

“*It was the aim of classical physics eventually to resolve natural phenomena into a play of elementary units governed by ‘blind’ laws of nature. This was expressed in the ideal of the Laplacian spirit which, from the position and momentum of particles, can predict the state of the universe at any point in time. … In contrast to this mechanistic view, however, problems of wholeness, dynamic interaction and organization have appeared in the various branches of modern physics. … It is necessary to study not only parts and processes in isolation, but also to solve the decisive problems found in the organization and order unifying them, … Again, similar trends appeared in Psychology. While classical association psychology attempted to resolve mental phenomena into elementary units—psychological atoms as it were—such as elementary sensations and the like, gestalt psychology showed the existence and primacy of psychological wholes which are not a summation of elementary units and governed by dynamic laws.” (Von Bertalanffy*, *1969/2009**, p. 31)*.

This paragraph articulates what has become a core tenet of systems theory which is that, from the interaction of the lower order constituent elements of a system, *an entirely new form of organization emerges*, one which cannot be fully explained by the basic principles of interaction of the constituent elements, even though it emerges from their interaction (Haken and Levi, 2012). This process of emergent self-regulation is most fully described by the concept of recursive organization in systems theory, which is described next.

## Recursive epistemology in systems theory

The concept of recursion is used in number of fields, and has slight variations in its meaning across some of these different fields. Within mathematics and computer science a recursive function is one whose term involves calling itself, with each successive application of the function referred to as an “iteration” (Shoenfield, 2001). Within the broad fields of systems theory and cybernetics, the term has also been used in different but related ways by Bateson (1978), Beer (1972), Keeney (1983), and Maturana and Varela (1980).

As described by Keeney (1983), a primary assumption of recursive organization of systems is that a system may be described as having different levels of organization of its activity. A second assumption is that the organization at higher-order levels influences the activities at lower levels of description. Keeney gives the example of how one might view a dance between two partners as recursive levels of organization. For example, the first partner in the dance may step to their right; this basic level of behavior could be considered the lowest level of organization in the current scheme. However, a higher level of organization refers to the level of interaction: the first partner steps to their right, while the second partner steps to their left. The activities at the level of behaviors are subordinated to this level of interaction. Yet a higher level of organization is at the level of choreography or pattern of interaction: the dance is a waltz. The activities at the level of behavior as well as interaction are subordinated to this pattern of choreography.

Keeney (1983) uses this formulation to describe a problematic pattern of marital interaction. The husband says he nags because the wife withdraws, while the wife says she withdraws because the husband nags. However, we might understand the behaviors (nagging and withdrawing) as being subordinated to a pattern of interaction which might be stated as withdraw, nag, withdraw, nag, withdraw, nag, crisis, reset (the pattern could equally begin with “nag” instead of “withdraw”). This systems-based formulation which indicates that behaviors within relationships are organized by stable patterns of interaction has come to have very strong empirical support over the decades-long work of John Gottman and colleagues in marital interaction patterns (Gottman et al., 2002).

Beyond these first two assumptions, a further assumption of recursive epistemology refers to the idea that the higher levels of organization of the system emerge from the activities at lower levels of organization. Similar to the earlier example of the “pecking order” of chickens emerging from the behaviors of pecking (Grobbelaar, 1989), so the pattern of marital interaction described above may actually emerge from nagging and withdrawing behaviors to begin with. However, the pattern becomes self-organizing over time, and begins to organize the nagging and withdrawing behaviors, so that they come to have a predictable pattern.

A final proposition of recursive organization is the principle that though higher levels of organization come to define the activities at lower levels, they cannot violate the principles of organization of those lower levels. In other words, the pattern of marital interaction of nagging and withdrawing that emerges between the marriage partners cannot consist of behaviors or emotions that the partners themselves are not capable of producing. The pattern of activity that emerges from the behaviors and interactions of a system's elements must lie within the parameters of possible behaviors or states of the system and its elements (Grobbelaar, 1989). In other words, while the higher-order levels of organization come to dominate the activity of lower-order elements, it cannot violate the lower-order principles of organization of those elements, nor exceed the range of potential actions of those elements.

Such a notion of emergent self-organization has received tangible support from a range of research trajectories. Hermann Haken developed a model of self-organization of coherent laser light, and how this self-organizing shift distinguishes it from non-coherent light. Haken's model, and the theoretical field it has given rise to (Synergetics) has become an established research trajectory across several disciplines, including biology (the “swarm” intelligence), computer engineering (AI studies) and molecular robotics (Haken and Levi, 2012).

In his book entitled “Reality is not what it seems,” Rovelli (2016) of the Centre de Physique Theorique in Marseille, points toward the long-standing problem in physics which is the apparent fact that principles of physics which hold true at the macroscopic level of general relativity, do not hold at the microscopic level described by quantum mechanics, and vice versa. Though substantial efforts in the field of physics have attempted to bridge these two levels, no satisfying solutions have yet been found. Rovelli shows how research into loop quantum gravity has suggested that when basic subatomic quanta of gravitational fields cluster together in complex relationships, these aggregates start to interact with one another and develop novel behaviors that are unique to that level of aggregation. Rovelli shows how such aggregates can be described as occupying different levels of organization, and argues that quite profound changes occur once quanta aggregate up to the level of curving spacetime, meaning that activities at that level simply cannot be predicted by principles obtaining at subatomic levels alone, though they do emerge from activities at those levels (Rovelli, 2016).

The key implication that this view of recursive organization has for systems, is that a complex system may be understood as consisting of a number of levels of organization, each of which organizes the structure and therefore behavior tendencies of the system. These layers are hierarchical, in that the highest level has the greatest influence over the behavior of the system though it remains constrained by the lower levels, and all the levels are always operative, and not in competition with one another. A last point is that the developmental history of the system indicates that each successive layer of organization emerges from the layer below (which is why it cannot violate the principle of organization at the lower layer), and then *entrains* that subordinate level such that the subordinate regulatory principles now come to serve the superordinate ones, which now have a greater influence over the system's further behavior (Keeney, 1983; Grobbelaar, 1989).

For example if we adopt a two-level scheme which consists of the principle of natural selection (or survival of the fittest) and a second principle which is that culture exerts an organizational influence on the structural development of the person (especially the brain), we could think of the cultural organization as superordinate and the influence of natural selection as subordinate. In other words, we could understand the rise of cultural organization as emergent from organization through natural selection, due to the survival advantages bestowed by group membership and communication. However, over time and development, the influence of culture becomes self-organizing such that its influence is not fully explained by the principle of natural selection, and (as the superordinate emergent principle) it comes to have a greater influence over the regulation of the system: while our daily behaviors may (hopefully) mostly have the tendency of enhancing our survival, they are more specifically shaped by cultural information and norms. However, the principle of natural selection continues to operate: as long as there is any pattern to who survives and reproduces, evolution continues to take place. Further, we could say that the principle of natural selection becomes entrained by the influence of culture, such that our evolution comes to be influenced more and more in the direction of adaptation toward a cultural environment (a study of the cultural influence on evolution can be found in Richerson and Boyd's, 2006 work “Not by Genes Alone”). Armed with this concept of hierarchical recursive organization (referred to as HRO for the remainder of the text), we now move toward exploring a central difficulty in the historical development of psychoanalytic theory.

## Freud's “project” and the self-regulation of the nervous system

Though Freud was not influenced by the growth of systems theory, in his posthumously published work “The Project for a Scientific Psychology” (1950), he set himself a task that was very similar to that prescribed by von Bertalanffy. In “the Project” his stated task was to demonstrate that all principles of human behavior, affect and psychical activity were determined by the interaction of neurons (the “constituent elements” of the psychical system, in his view), and the influence they exerted on one another through an energy he termed “Qn”:

“*The intention is to furnish a psychology that shall be a natural science: that is, to represent psychical processes as quantitatively determinate states of specifiable material particles, thus making those processes perspicuous and free from contradiction. Two principal ideas are involved: (1) What distinguishes activity from rest is to be regarded as Q [referring to the term ‘quantity’ described below], subject to the general laws of motion. (2) The neurones are to be taken as the material particles.” (Freud*, *1950**, p. 295)*

He then proposed that the nervous system is regulated by a single organizing principle (he called it the primary principle), which is to divest itself of this energy (Qn), which he called a principle of *inertia*, though elsewhere referred to it as a principle of constancy. He tried to explain instances of the nervous system refraining from discharging energy as the result of the influence of a secondary principle, serving the interests of behaving adaptively with regard to the environment.

The important consideration here about “the Project” is the fact that he very clearly wanted to generate an entirely “bottom-up” description of the self-regulatory activity of the nervous system. Through defining different types of neurones and the types of influence (and barriers to influence) they exerted on one another, he hoped to explain the entirety of operation of psychical processes as complex as consciousness, memory and attention purely through these basic energic interactions of different types of neurons. He expressly avoided describing any process that could not be traced back to this basic interaction of different types of neurons (Connolly, 2016).

The similarity between the aims of “the Project” and the principles of the growing systems theory field of cybernetics (self-regulation) were remarked upon by Strachey in his translator's introduction to the text:

“*It has been plausibly pointed out that in the complexities of the ‘neuronal’ events described here by Freud, and the principles governing them, we may see more than a hint or two at the hypotheses of information theory and cybernetics in their application to the nervous system. To take a few instances of this similarity of approach, we may note first Freud's insistence on the prime necessity for providing the machine with a ‘memory’; again, there is his system of ‘contact-barriers,’ which enables the machine to make a suitable ‘choice,’ based on the memory of previous events, between alternative lines of response to an external stimulus; and, once more, there is, in Freud's account of the mechanism of perception, the introduction of the fundamental notion of feed-back as a means of correcting errors in the machine's own dealings with the environment.” (Strachey in Freud*, *1950**, p. 292–293)*

However, Freud failed in this endeavor. Once he discovered that he could not overcome a number of internal contradictions in the system he had designed, he abandoned the project, and tried to have it suppressed, later stating:

“*I can no longer understand the state of mind in which I hatched out the ‘Project”' (Freud*, *1950**, p. 285)*

This failure appears to have been significant, in that from this point on, Freud appeared to begin to accept the use of top-down principles of regulation of the psyche for which he had not been able to generate a “bottom-up” explanation. This is really the start of his distinction of the “psychological” theory from neurology, in which he sought to describe principles that organized psychic life, even though he could not offer a description on how those principles emerged from its organic base:

“*I have no inclination at all to keep the domain of the psychological floating, as it were, in the air, without any organic foundation. But I have no knowledge, neither theoretically nor therapeutically, beyond that conviction, so I have to conduct myself as if I had only the psychological before me” (Freud*, *1898/1985**, p. 26)*

A good example of this appears in his next major text which was “The Interpretation of Dreams” (1900/1991). In it, Freud introduces the concept of a preconscious gate, which limits access to consciousness of psychic material on the basis of whether the discharge of their energy causes pleasure or unpleasure, though no adequate physiological description of that pleasure or unpleasure is articulated in the text (Grobbelaar, 1989; Connolly, 2016).

Freud's effort in “the Project” sought to describe bottom-up processes that generated the self-regulation of the psyche and behavior, and in this respect bears similarity with the aims of general systems theory. However, Grobbelaar (1989) has argued that the failure of Freud's theorizing in this regard was not due to a lack of effort or diligence, but due to the lack of an adequate systems-based epistemology.

Referring back to the earlier quote by Von Bertalanffy (1969/2009) regarding the need to study emergent principles of holism and organization, not just elementary units moved by “blind” or mechanical laws of nature, this same point can be made with regard to Freud's project. If we agree with von Bertalanffy, we might suggest that the primary reason Freud's “project” failed was because he tried to explain the operation of the system based entirely on the basic principles of operation of the base elements (his description of specific types of neurons and the energy transfer between them). Essentially, we might say that he failed because he did not recognize the core insight of systems theory and HRO, which is that the basic energic interactions between types of neurons that he described in “the Project” should give rise to an entirely new form of (superordinate) organization, not fully explained by the basic energic interactions he described. We might agree with Freud's idea in “the Project” that the principles that organize the psyche and behavior might emerge from the more basic principles of energic interaction of neurons, but rather we should not agree that they can be fully explained by the principles governing that basic interaction.

## The example of the pleasure principle and psychic energy

To illustrate the importance of this distinction, a good example might be that of the pleasure principle in psychoanalysis, which is the tendency of the psyche to maximize pleasure and minimize unpleasure (Freud, 1911/1963). It is important to note that the pleasure principle appears to operate as a relatively fundamental principle ordering human behavior and psychic life, and so we might think of it as an important “top-down” or superordinate form of regulatory principle. However, from the beginning of his theorizing about the pleasure principle, Freud attempted to generate a bottom-up explanation for it through basic processes of energic interactions of neurons, with his theory of psychic energy. In “the Project” (1950), he initially stated that a discharge of energy from the neurons was pleasurable, while an “accumulation” of energy was unpleasurable. However, after the difficulties met in “the Project,” this link between pleasure and energic principles already began to fray in chapter 7 of “The Interpretation of Dreams” (1900/1991) where Freud suggested that discharges of energy could sometimes also be unpleasurable to the psyche and the preconscious gate somehow became the decisive process that allowed pleasurable discharge but opposed unpleasurable discharge, though as stated above, Freud could not offer a bottom-up physiological description in that text for how the preconscious gate might make that distinction. Freud (1920/1955) returned to this problem in “Beyond the Pleasure Principle” where he defined bound and unbound states of energy (cathexis) but in the same paper he questioned whether pleasure and unpleasure could be defined in terms of bound or unbound energy. In that paper he then made an interesting suggestion that pleasure may be linked to the *rate of change* of discharge, but never developed that idea further in his work (though the reader is referred to an exploration of this topic from the FEP perspective, where the intensity of emotion as well as its positive or negative valence, is linked to the rate of change of free energy, found in Joffily and Coricelli, 2013).

Thus, the failure of adequately linking the pleasure principle with energic processes may be an example of the problem defined above, in that the pleasure principle may emerge from basic energic interactions between neurons but can't be fully described by these. The same can be said for the energic theory itself. Freud had high hopes for his energic theory: in “the Project” (1950) he claimed that the tendency toward discharge was the fundamental motivation of all thought, emotion and behavior, and energic principles are recognized as a core metapsychological foundation of psychoanalysis (Rapaport and Gill, 1959). However, after his difficulties in describing energy as a neuronal physiological quantity in the project, he no longer attempted to describe it in terms of cathexis of Qn in neurons, despite continuing to use concepts of cathexis, binding and discharge for much of his career. Like the pleasure principle, energic concepts became described as “top-down” organizing principles of the nervous system that were not adequately described in terms of how they emerged from the basic interactions of the nervous system elements.

A central purpose of this paper is to demonstrate how useful the concept of HRO can be in linking these bottom-up and top-down levels, and indeed, this concept can address this problem of the emergence of organizing principles such as the pleasure principle. We could reformulate our definition of psychoanalytic principles of regulation of the psyche such as the pleasure principle (or another like it) as a recursively higher level of organization of the nervous system, that must nonetheless emerge from the basic interactions of the nerves themselves. While we might say that the pleasure principle (as formulated by Freud, 1911/1963) cannot violate the basic principles of organization of the nerves and their interaction, it also cannot be adequately modeled by those basic principles of interaction. This difference of organizational levels is proposed as the key reason for the failure of “the Project” (Freud, 1950), as well as the difficulty faced by Freud throughout his career (and by many subsequent psychoanalytic writers) to link the principles of organization of the psyche with those of the basic interactions of the nervous system. Had Freud been armed with a recursive epistemology, he would probably not have tried to write “the Project” or rather, may have taken a different approach to the material.

## Recursive epistemology and the problem of different principles of organization at physical and mental levels

Beyond this example of the pleasure principle and basic interactions of neurons, it can be stated that the underlying problem is really a deeper one which is the relationship between the principles which organize the structure of the body (including the nervous system) with those that appear to regulate the mind, and subjective experience. We could restate this particular aspect of the mind-body problem as a statement that the mind occupies a higher (superordinate) level of recursive organization in the person than the body does. However, this statement by itself doesn't add much to our understanding, beyond implying certain assumptions about the superordinate/subordinate relationships between the levels. What is needed is a more specific analysis of the principles of organization occurring at these different levels and defining a process whereby the emergence of the recursively higher level is explained.

The idea that mind and body, or mind and nervous system, occupy different levels of organization of the same system is not a new idea in psychoanalysis; a number of authors have not only expressed such a viewpoint but also attempted to reformulate some core psychoanalytic concepts from this viewpoint, notably including work by Grossman (1992), Seligman (2005) as well as Rosenblatt and Thickstun (1970, 1977, 1984). However, despite these efforts, systems theory epistemology, and recursive organization in particular, has never gained meaningful visibility in the mainstream of psychoanalytic (or neuropsychoanalytic) thinking.

However, the rapidly growing interest in the FEP may indeed demand a better understanding of these systems concepts from those members of the psychoanalytic community interested in the FEP. Friston's FEP is so important to psychoanalysis because it reflects a critical step forward to solving the problem of differing principles of organization at neural and psychological levels. It is the purpose of the present paper to demonstrate how this is so, as well as the necessity of a concept of recursive organization in order to make sense of the level of organization that Dr. Friston's work belongs to, and a correct understanding of its relationship to the level of constructs central to psychoanalysis. This is necessary not only to recognize the powerful potential that the FEP has as a core metapsychological principle within psychoanalysis, but also to avoid overstating its role, and recognize the limitations the principle has for application within psychoanalysis as well. In order lay the groundwork to clarify this role of the FEP in the psychoanalytic scheme, we will clarify what is meant by a recursive description of the psyche, by following the indications expressed by Grobbelaar (1989).

## Recursive levels of organization in psychoanalysis

Grobbelaar (1989) stated that:

“*…the view which is currently maintained by convention can be seen to constitute some sort of hierarchy with at its lowest level the inorganic domain, at the next level the organic, and finally at the highest level, the informational domain (Stoker*, *1969**). Although the components and their properties differ from one level to the next, the person as a system is constituted by the relations which obtain between the components at the same level as well as between components on different levels which defines the person as a unity. Furthermore it is clear that the organization at the lowest level sets the parameters for the recursive ordering of components/elements at the next level, so that the organization at the inorganic level will be reflected in a general way at the organic level, and in an even more indirect way at the informational level…Freudian theory is an attempt to identify the common human patterns at the inorganic and organic levels which determine the informational (psychological). … Freudian theory furthermore hypothesized that, at the inorganic level, the principles of organization which emerge from the energic interactions are the tendencies towards tension reduction and homeostasis, which are reflected at the organic level as the pleasure principle, and at the psychological level as the hallucinatory wish-fulfilment and the process Freud described as censorship.” (p. 134–136)*

What Grobbelaar is attempting to do in this quotation is show how the processes at the psychological level are founded upon processes at work on the organic level of organization which are themselves founded upon processes at the inorganic level. It should be noted that it is not inevitable that these three levels should be used to describe the human as a system. Bateson (1978) suggests an infinite regress of levels, and that the observer selects the levels of description. However, besides selecting these for the purposes of convention, these three levels are significant, precisely because they appear so different in their organization: organic life appears to be so different from the inorganic matter we observe around us, and human consciousness in turn seems so markedly different from the self-regulation of most biological organisms, though that difference might be less marked than we believe in many cases.

What such a recursive description of the organization of the human organism necessitates, is a theoretical perspective that shows how regulatory principles of the psychoanalytic mind are related to the regulatory principles of biological self-organization at the organic level, which themselves should be related to regulatory principles at an inorganic level. The key proposition of this paper is that Friston's FEP (working in concert with evolution through natural selection which is discussed later) represents a fundamental principle of biological self-organization which has not only been shown to have consilience with fundamental propositions of psychoanalysis (Hopkins, 2012; Connolly, 2016), but which is shown to be founded upon regulatory principles at the inorganic level as well (Friston and Stephan, 2007; Friston, 2013).

**Figure 1:**
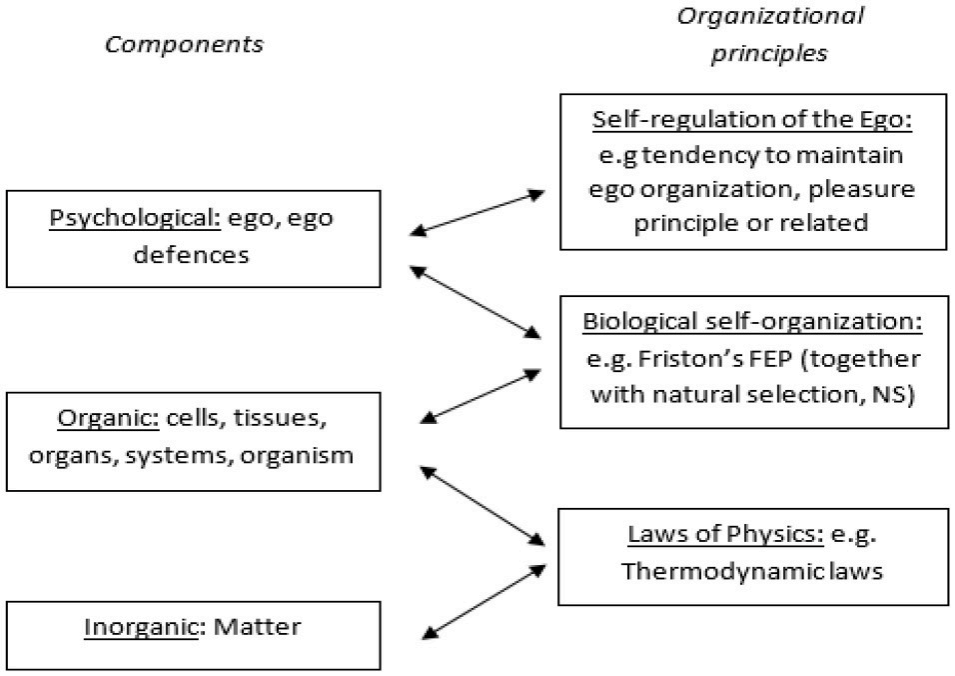
Three levels of recursive description of organization of psychical phenomena.

Figure 1 presents a three-level recursive description of psychoanalytic regulatory principles that is influenced by Grobbelaar's (1989) description (and a similar model found in Connolly, 2016), which demonstrates this hierarchical relationship. A brief narrative description of the diagram would start from the bottom, and run as follows: inorganic elements (atoms and molecules), interact with one another (within the constraints of thermodynamic principles), and come to generate a new form of organization which is organic, or biological self-organization (which operates within the constraints of the FEP, itself constrained by natural selection, described later). The predictions encoded within the organization of the body (and after an evolutionary step, the nervous system) eventually come to be “aggregated” in the sense of a self-organizing generative model, understood here to be the ego, which is regulated in turn by its own principles, such as the pleasure principle or following Connolly (2016), a tendency to maintain its own organization.

The bi-directional arrows are to indicate that the influence then becomes top-down as well, so that the behavior of body (including its inorganic components) come to be regulated by psychological level components, though in a way which does not violate the principles of FE and thermodynamics which regulate the lower levels.

The place of Friston's FEP in this scheme is to formalize biological self-organization, which means it provides a constraint within which all informational exchange processes within an organism take place (Friston, 2012). As a result, the level above (which is psychological) cannot violate the FEP. However, as will be discussed next, new organizational principles emerge at this level, so that it is not fully explained by the FEP. These principles are elaborated upon in the next few sections.

## From inorganic foundations to biological self-organization

While an engagement with levels of organization within the inorganic realm is beyond the scope of this paper, thermodynamic principles are nonetheless significant at the level of matter at which organic life occurs, and a discussion of their relationship with biological self-organization can be found in Friston and Stephan (2007). Their text states that biological systems are special in the natural universe because they appear to violate the second law of thermodynamics which is the tendency toward entropy: biological systems seem to show increasing levels of organization in their development rather than a tendency toward entropy. However, this apparent violation is just apparent—biological systems do not violate the second law but rather display a form of organization (the FEP) that is akin to the fluctuation theorems that underlie stochastic thermodynamics. These generalize the second law. As we will see later, the FEP is effectively an example of Hamilton's principle of least action. In order to develop an understanding of Friston's FEP of biological self-organization, a related verbal model (from Maturana and Varela, 1980) is offered next.

Humberto Maturana, a Chilean biologist was once asked by a student*: “‘What began three thousand eight hundred million years ago so that you can say now that living systems began then?”’* (Maturana, 2002, p. 6). He realized that to answer it, he would have to identify what a living system is, and what makes it a living system. In trying to answer the question, he first made the assumption that living systems were “closed” in the sense of having an operational boundary (though being thermodynamically “open”). He then focused on the circularity of the basic metabolic reactions:

“*… nucleic acids participate with proteins in the synthesis of proteins, and that proteins participate as enzymes with nucleic acids in the synthesis of nucleic acids, all together constituting a discrete circular dynamics. … As I was drawing a diagram of this circularity, I exclaimed ‘This is it! This is the minimal expression of the circular closed dynamics of molecular productions that makes living systems discrete autonomous molecular systems.”’ (Maturana*, *2002**, p. 7)*

From this insight, together with Varela, he coined the term “autopoiesis,” which means “self-making.” In other words, what constitutes a living system is two conditions. First, a boundary that creates a closed autonomous molecular structure (despite being thermodynamically open to flow of molecules in the environment). Second, a process of self-making, where a dynamic circular process takes place in which the components of the process build or maintain a structure which in turn generates the components. While this autopoietic system is closed in the sense of its organization, it is nonetheless open to the flow of molecules in the environment which participate in this self-making, and without which it would cease (Maturana and Varela, 1980; Maturana, 2002). This dependence of the autopoietic organization on the conditions in the environment led him to coin the term structural coupling.

Maturana defined living systems as structure-determined systems, in that their behavior is determined by their structure. However, that structure is occurring within a medium (or environment) and is recursively constituted from moment to moment by interactions with that medium, such that a change in one must imply a change in the other (Maturana and Varela, 1980; Maturana, 2002).

It could be said of the FEP that what it formalizes is the structural coupling of autopoietic systems. In the following section, the FEP is briefly introduced in a manner to highlight its similarity to a concept of structural coupling as a principle of biological self-organization.

## Friston's FEP as structural coupling

The FEP describes how a range of biological phenomena unfolding over time can be described as the minimization of the error between the predictions afforded by a generative model of the causes of a living system's inputs and the inputs being predicted. To state it in another way, the organism's structure encodes a model of its environment (the generative model), and over time, this generative model should become a better and better predictor of the system's inputs (instigated by the environment). The minimization of this error term can be accomplished through two routes, one being the “Bayesian” updating of the generative model (to provide better error resolving predictions), the other being through the system taking an action to alter the inputs, and thereby bringing the system's sensory samples in line with predictions. While the bulk of the research that Friston and colleagues have done with the FEP have focused on describing neurophysiological processes, the principle is understood as having applications well beyond neurophysiology, and applicable to a broader biological science, including being applicable to organisms without nervous systems (Friston, 2010b, 2012).

From the above definition, a strong consilience can be seen between the FEP, and Maturana's formulation regarding structural coupling, where the structure of the organism is continuously constituted through recursive interactions with the environment. Further, Friston's FEP has shown to provide a basis for modeling Maturana's concept of autopoiesis itself. In a paper entitled “Life as we know it,” Friston (2013) shows how the formation of living systems (including a simple form of autopoiesis) can be modeled using free energy minimization with only two assumptions.

The first assumption is ergodicity, which is that, over a sufficiently long time-period, the average amount of time a system spends within an accessible state is proportionate to the probability of finding the system in the state. In the language of random dynamical systems, this is equivalent to saying that the system has a random dynamical attractor; namely, a set of states that the system frequents with a high probability. The second assumption is the existence of a “Markov blanket,” which corresponds to the boundary described above: Friston (2013) states that should a boundary exist where relations on one side are conditionally independent on influences outside the boundary, this boundary will come to constitute a Markov blanket through which the internal states of a system exchange with external (environmental) states. This exchange can be formulated as an influence of the environment (external states) on the system (internal states) that is mediated by sensory components of the Markov blanket. Conversely, the influence of the system (internal states) on the environment (external states) is mediated by the active components of the Markov blanket. Note again the emergence of circular causality that has all the hallmarks of a perception and action cycle.

The existence of the Markov blanket (that separates the system from the environment in which it is immersed) implies ergodicity of the entire partition (into external states, internal states and their Markov blanket). In turn, this requires the internal states to minimize free energy as a necessary condition for the preservation of the Markov blanket. The particular aspect of free energy minimization, from the perspective of psychology and psychoanalysis, is that free energy is not just a function of states, it is a function of the probability distributions of Bayesian beliefs that are entailed (i.e., encoded) by internal states. Technically, this means the free energy is a function of a function or a functional. The key issue here is that the imperatives for self-organization are now framed in terms of probabilistic inference and beliefs. This follows from the fact that the free energy provides a proxy or bound approximation for Bayesian model evidence. Put simply, minimizing free energy necessarily maximizes the evidence for a system's (generative) model of environmental (external) states^1^. Given the circular causality above (e.g., action perception cycles), this means any system with a Markov blanket will appear to gather evidence for its own existence; which has been called self-evidencing (Hohwy, 2016)—and is closely related to early theories of self-organization such as the good regulator theorem (Conant and Ashby, 1970).

It is evident that this notion of a Markov blanket is, formally, very similar to Maturana's assumption of a closed boundary condition for the formation of a living system. From this, Friston concludes that the formation of living systems is almost inevitable in a universe that provides ergodicity and the existence of Markov blankets. Such blankets (surrounding open self-organizing processes) may be ubiquitous in the universe but the Markov blankets associated with living systems may have a particular form of hierarchical self-assembly that corresponds to the *self-making* referred to by Maturana (2002), rather than just *self-organization* which occurs throughout the natural universe.

The claims made by Maturana are not precisely the same as that made by the FEP, and are a verbal principle rather than the precise mathematical principle that is the FEP. However, Friston et al. (2015) have suggested that active inference can be viewed as a formalization of autopoiesis. The present purpose of drawing parallels between Maturana's theory and the FEP is to present an accessible verbal principle related to the FEP which allows its place within a recursive description of the mind to be perceived. From this section, it is hoped that the reader can see that what is most precisely described by the FEP is a process of biological self-organization, rather than being a purely “neurocentric” principle (Friston, 2010b), and for this reason it is presented as a fundamental constraint organizing organic systems in the current description.

## The role of evolution through natural selection

A key question that might be raised at this point is the role of evolution through natural selection (NS is used henceforth). Thus far, the paper has presented the FEP as the key principle of organization of biological systems, despite a wealth of evidence suggesting that the structure of organisms including the nervous system and human behavioral tendencies have been shaped by evolution through natural selection (Cartwright, 2016). For this reason, care is taken to try to explain the relationship between the FEP and NS adopted by this paper and the useful role that HRO can have in clarifying this relationship as well.

Within the biological realm, where the FEP is operative, HRO of biological systems becomes constrained by the FEP, such that the FEP may be described as a superordinate organizational principle which entrains (and constrains) HRO, resulting in further hierarchical organization being reflected in the structure of the organism, and most relevantly, the hierarchical organization of the structure of the brain, such as the layers of the mammalian cortex. The role of NS can be considered superordinate to the FEP in a similar way. In essence, once organisms are characterized by a cycle of life, reproduction and death—and provided there is some measure of environmental order influencing selection of survival and reproduction—survival of the fittest comes to act as a recursive feedback loop slowly operating at a population level, through each generational iteration. This results in a new organizational principle entraining the operation of the FEP (and thereby HRO as well), such that the organic phenotypes that exist now reflect this influence by NS. At the same time, the NS principle cannot violate the FEP, and as suggested by Hobson and Friston (2016), evolution can be understood as minimizing the free energy of specific phenotypes. As such, the different organizing principles of FE and NS are understood here as not in competition with one another, and though hierarchically arranged, all operate in organizing the organism and its behavioral tendencies^2^.

Regarding the relationship of NS with the regulatory principles of psychoanalysis, connections have been made between the level of organization in psychoanalysis with that of natural selection (Hopkins, 2003, 2004, 2015; Hopkins, “Group conflict and group violence: a perspective from Freud and Darwin,” forthcoming). A good example is found in Hopkins (2004; Hopkins, “Group conflict and group violence: a perspective from Freud and Darwin,” forthcoming), where he describes an organizational principle emerging from natural selection, which is the tendency toward outgroup aggression, which shows how the survival advantage granted by group identification (and outgroup aggression) may underlie the evolution of mechanisms of projection and introjection described by psychoanalysis.

This description of the relationship between the FEP and NS requires much more detailed discussion than is given here. However, this paper focuses on the specific role played by the FEP in the hierarchical self-organization of the organism, and particularly its relevance as a foundation of psychoanalytic principles of self-regulation. This specific and unique importance of the FEP lies in how it constrains HRO in a scale-free manner within a recursive hierarchy of levels of organization in the brain, and the nature of message passing between them, which is addressed later.

## The scale-free nature of the FEP in the behavior of biological systems

The concept of a scale-free principle is one that applies to all possible levels of scale of a phenomenon at hand (Mitchell, 2009). In other words, we would say that the principle holds no matter the scale at which you observe a phenomenon. What this means for the FEP, is that no matter at what scale you observe biological systems, the FEP should not be violated. In other words, the FEP can be observed at the level of single cells, or any components they are made of (e.g., mitochondria, dendrites), at the level of organs, systems and whole organisms (Friston, personal communication, 13th July 2015). At a neural level alone, the FEP may apply over a short time span to the activity of neurons, and over a longer time span to the reorganization of neural connections (Friston et al., 2006).

A large number of empirical findings have begun to show the variety of phenomena that can be described using the FEP formulation. These include the hierarchical deployment of cortical areas, neuromodulatory gain control and associative plasticity, receptive field effects, components of evoked cortical responses, and on a cognitive level, perceptual categorization, temporal sequencing and attention (Friston, 2010b). Such research is ongoing, and it is likely that this is just the beginning, and that there will be a substantial increase in phenomena described by the FEP, over the next years.

This apparent scale free perspective supports the notion of HRO adopted in this text, as it suggests that any hierarchically superordinate forms of organization that may develop within biological organisms, should nonetheless not violate the basic organizing principle of this organic level which is the FEP. Just as there is no action a human system can take which violates the principles of thermodynamics, so there is no action a human system can take which (viewed over a sufficiently long period) can violate the FEP. If you knew exactly what to measure and how to measure it, you could show that a person's action or thought always minimizes FE, at least when averaged over an adequate time period (technically, the average of an energy is known as a Hamiltonian action; this means that the free energy principle is a statement of Hamilton's principle of least action). Though it may be that a human system does something that appears to raise the overall level of FE in their system in the short term, the effect may be compared to dropping a ball and the principle of gravity: when it bounces and travels upwards it appears to violate the gravity principle, but over time, it will obey the principle (a similar comparison for the principle of psychic energy was found in Galatzer-Levy, 1983).

## Can all human behavior be modeled by the FEP?

The above section would seem to imply that all the behavior comprising a human living system is subject to the FEP, which is indeed correct. However, while it could be claimed that the FEP as a working principle is not violated at any levels of organization of the human system (above the inorganic), this does not mean that all phenomena in a human living system are appropriately modeled using the FEP. This distinction can be displayed with an analogy.

Newton's second law of motion, force equal mass times acceleration (F = ma), should apply to the movement of all physical bodies in space, within particular limits in terms of mass, gravity and so on. However, if an engineer was supposed to model the complex operation of forces moving through the structure of a jet airplane as it flies through atmosphere, armed only with the model F = ma, it would prove to be a wildly impractical task. This would require a complete knowledge of every vector of force at work on and within the structure of the airplane at every moment, as well as perfect theoretical knowledge of how those vectors will operate from moment to moment. In other words, our engineer would need additional principles, in the form of models of “aggregate” processes such as lift, drag, stress dynamics, turbulence and others. These aggregate models involve different equations than that of F = ma, though none of them can violate this foundational model.

For human behaviors at the level of interest of psychoanalysis (for example actions, speech, thoughts, dreams and so on), the situation is comparable. While the previous section has described how human behavior and psychological processes at all levels cannot violate the FEP, if one were expected to model the complexity of human behavior and thought armed only with the FEP equation, it would be an equally wildly impractical task. One would need to know to know the exact state of activity of the entire nervous system (and indeed the entire body), as well as a comprehensive range of precise theoretical principles for how this state will progress from moment to moment (including how these states relate to thoughts, emotions and behaviors at the observable level). Just as in the analogy of the jet aircraft above, one would need to have a range of additional principles that model such aggregates of activity that hold at the level of interest.

The view of this paper is that the propositions of psychoanalytic theory (such as transference, repression or splitting) reflect such models at this higher level of organization, similar to lift, drag and turbulence in the engineering analogy. In the long term, the challenge is to demonstrate the relationship of these models to the foundational organization of the FEP through a recursive description of the levels of organization superordinate to the FEP, up to and beyond consciousness. This task is returned to later in this paper.

However, a question the reader might have at this point would be to ask how levels of organization of the human system that are superordinate to the FEP, can be subject to the FEP without being sufficiently modeled by the FEP. It is hoped that these two sections have shown that it is no contradiction at all. While there is nothing a human can do that can violate the second law of thermodynamics, that law is hardly enough to model human behavior. While the organizational principle of the FEP is much closer to the level of interest that is psychoanalysis, the same limitation applies. The FEP does nonetheless retain some influence over superordinate levels through feedback loops, throughout the levels of recursion.

## Friston's FEP as a model of recursive organization of hierarchy in the nervous system

**Figure 2:**
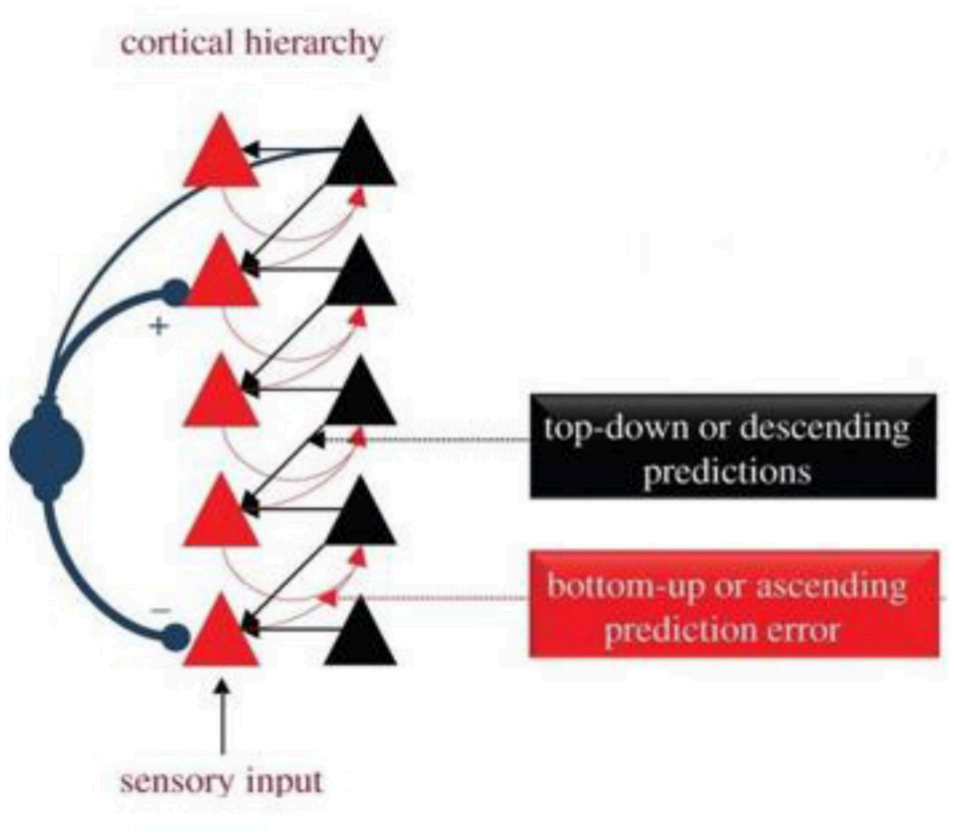
Hierarchical organization of message passing in the brain, from Seth and Friston (2016). This illustrates the recursive message passing it implied by predictive coding formulations of free energy minimization; where in prediction errors are passed up the hierarchy and predict ions are sent down to cancel prediction errors. The blue circles and arrows denote precision that controls the influence of ascending predict ion errors. Here, precision corresponds to the inverse variability or confidence assigned to prediction errors.

The diagram in Figure 2 below demonstrates a hierarchical and recursive description of organization in the nervous system found in predictive coding formulations of Friston's FEP, whereby surprise or prediction error messages progress “upwards” through the hierarchical generative model to successively higher levels of abstraction, which respond with “downwards” predictions.

As stated earlier, Friston (2010a) has suggested that higher level predictions refer to increasing levels of abstraction. As suggested in Hobson et al. (2014):

“*Many of the interesting insights offered by equating consciousness with the process of inference rest on the hierarchical nature of generative or virtual reality models. In hierarchical models, inference can be decomposed into multiple levels, with progressively higher or deeper levels of representational abstraction or explanation. This leads to the distinction between inferences at low levels of sensory hierarchies—that can be associated with unconscious inference in the sense of Helmholtz (**1866/1962**)—and at higher levels that could be associated with conscious percepts and concepts. Consider now a further hierarchical level that predicts (and selects) the particular trajectory that is enacted. This level may generate top-down predictions of proprioceptive trajectories and their visual consequences. In other words, we have moved beyond simple motor representations to a hierarchical level where expectations (neuronal activity and their associated beliefs) are quintessentially sensorimotor in nature. At this level, the multimodal nature of descending predictions (aka corollary discharge) renders the expectations amodal. Would these constitute conscious experience? One could argue that these high-level, dynamically structured beliefs are much closer to phenomenal consciousness. Furthermore, if we now equip our hierarchical model with models that distinguish between the consequences of self-made acts and the acts of others, we start to get closer to conceptual expectations of the sort that may underlie subjective consciousness.”*

This account suggests that the predictive model is organized at multiple nested layers, all of which are influenced by the FEP through this recursive feedback process. As suggested earlier in this paper, the FEP comes to constrain HRO in the development of the structure of the human organism, and in the nervous system in particular, such that each recursively higher level of organization found in the body and especially in the brain, comes to have Markov characteristics, which implies that each level has self-organizing characteristics, and tries to minimize its own free energy. Note that this hierarchical nature of the generative model induces Markov blankets between different hierarchical levels, which mediate a circular causality through recurrent message passing between levels. The existence of a Markov blankets within the brain affords the opportunity for higher levels in the brain to make inferences about lower levels (c.f., metacognition, self-modeling and consciousness). However, while all these levels are influenced by the FEP through these recursive feedback loops, it is an error to suggest that the processes at all of these levels of recursion are fully explained by the FEP. A helpful example here would be natural selection. Although the principles of natural selection can be applied to all processes of biological evolution, simply knowing these principles does not help explain the emergence of particular phenotypic traits or constructs such as convergent evolution, speciation and other emergent properties such as selection for selectability (Kauffman and Johnsen, 1991; Kauffman, 1993; Knobloch, 2001; Frank, 2012; Campbell, 2016).

## The limits of the FEP in modeling consciousness and psychic experience

Friston (2008) has suggested that the multitude of nested levels of organization within the nervous system each have Markov properties, which implies that each level has some degree of self-organization. This also implies that each level would require additional principles of organization (beyond the FEP) in order to be adequately described. As consciousness occupies one of the highest levels of recursion within the organization of the brain (Hobson et al., 2014), all of these subsidiary nested levels (with their own unique organizing principles) would in turn influence the organization of consciousness. As such, the very high level of complexity involved in the organization of conscious experience is practically not able to be modeled by Friston's FEP equation.

This qualification seems important, as psychologists (including psychoanalysts) are often opposed to reductionist models of conscious experience, partly because they are so abstracted from the experience itself, but often more because reductionist models often simply cannot explain the complexity of their clients' experience. In this regard, they are entirely correct, as the previous paragraph has attempted to clarify. At the same time, however, it might be said that a therapist's perception of the complexity of their client and their lived experience should not be reduced by the acceptance that the client's psychic processes cannot violate the FEP, just as it should also not be reduced by accepting that client's body cannot violate thermodynamic laws. Extraordinary levels of complexity are possible within the broader constraints of thermodynamics as well as the FEP, which in turn require detailed analysis at the level of interest as well as the proximal influences of sub- and super-ordinate levels of description.

The generations of work in psychoanalysis to document the principles which seem to influence people's conscious experience and behavior, as well as the insights gained through clinical examination and self-reflection, are understood here as attempts to generate models of the organization of the phenomena of conscious and unconscious processes. These insights (and the models they represent) cannot be abandoned in favor of a far more foundational principle which is the FEP, for much the same reason as we should not abandon the use of the abstractions of integral calculus in favor of using the simpler language of linear algebra, to follow an analogy found in Rosenblatt and Thickstun (1984).

However, like Freud (1898/1985), we cannot afford to leave these insights “floating in the air” in a completely abstract theoretical space unconnected with any organic foundation. Following a call by Grobbelaar (1989), these models of experience and behavior at the psychoanalytic level of interest need to be reformulated within a new language that demonstrates the foundations of their organization within a recursive description, which has its foundations at the biological level.

The complexity of differentiation within the physical structure of the human body is huge, and there are also already a large number of models within the biological field that predict processes within this differentiated structure. Likewise, the differentiation within the brain is also highly complex. Following Bateson (1978), there are potentially infinite levels of regress in such descriptions, and it is neither possible nor even desirable to build a complete picture of every possible level of organic and neural organization superordinate to the basic level of biological organization which is the FEP, up to the level of interest which is here psychoanalysis. Rather, it is desirable to identify some of the most significant forms of organization that are foundational to psychoanalysis, but superordinate to the FEP, which can build an intelligible bridge between the two. The description provided by Grobbelaar (1989) provides a useful example of a recursive description of this kind which may illustrate a way forward.

## A recursive description of the representational organization of conscious experience

Grobbelaar (1989) offered a critique of Freud's account of the organization of consciousness (in terms of how psychic material does or does not become conscious), in that it did not offer a bottom-up recursive description:

“*As it stands now, Freud's formulation of the process of censorship defines it as an ad hoc defensive manoeuvre by one system, the ego, against another system, the unconscious, to stop dangerous elements (dangerous to the organization of the ego) from entering the ego. One should rather formulate from the bottom to the top, that is, in a theoretical sense. One should begin by defining the inherent qualities in the lower-order elements which … make it impossible for them to be taken up in a higher order system ….” (p. 142)*

He also states:

“*… the principles determining the perception of thoughts will be inherent in the thoughts themselves. Stated differently, if the organization of the ideational domain does not allow for the representation of certain ideas, thoughts or memories, then they cannot become conscious.” (pp. 139–140)*

In describing the principles inherent in thoughts which allow (or don't allow) them access to conscious, Grobbelaar (1989) refers to a comment made by Breuer in “Studies on hysteria” which refers to the notion that the quantity of affect attached to the thoughts, and the pleasure or unpleasure that that quantity of affect forms part of, determines their capacity to enter consciousness (Freud and Breuer, 1895/2004). This is related to Freud's notions that only sufficiently cathected thoughts or perceptions can enter consciousness (Freud, 1900/1991, 1950).

It can be noted at this stage that this determinant of the level of affect (or perhaps cathexis rather) has good consilience with Friston's (2010a) hierarchical description, which suggests that only information that is sufficiently surprising (or rather with sufficient gain, due to weighted precisions of surprise) can activate the predictions at the highest level of organization, which may be consciousness. Information that is insufficiently surprising is “automated” in the sense that it is sufficiently explained by predictions at lower hierarchical levels of the model, and does not elicit these higher-level predictions of consciousness (Hobson et al., 2014).

However, besides this requirement of the quantity of affect, Grobbelaar (1989) also points toward a comment made by Freud (1915/1957) in “The unconscious” where unconscious elements can only become pre-conscious through being connected with words:

“*The system unconscious contains the thing-cathexes of the objects, the first and true object-cathexes; the system Pcs comes about by this thing presentation being hypercathected through being linked with the word presentations corresponding to it.” (Freud*, *1915/1957**, pp. 200–201)*

Grobbelaar (1989) suggests that this formulation found in Freud (1915/1957) “The unconscious” is based on a much earlier paper on aphasia (Freud, 1891/1953) in which Freud suggests that a word-presentation is built up of a sound-image (auditory), the letter-image (visual), the motor-speech image (kinaesthetic) and the visual- and motor-writing image (visual and kinaesthetic) which become associated with one another through experience. Freud then states that the object presentation is built up in a similar way from kinaesthetic, visual and auditory experiences, and becomes linked to word presentation through associative learning, allowing for the object to reach conscious representation. The heart of Grobbelaar's argument is that this process can be described as a recursive one:

“*This process constitutes a recursive ordering of discrete elements of experience through successive acts of integration with new elements which progressively constitute the raw sense data at higher levels of psychological functioning.” (Grobbelaar*, *1989**, p. 147)*

Grobelaar's account of hierarchical levels of representation separated by boundaries here bears very strong similarity to a later paper by Grossman (1992) who also utilized Freud's paper on aphasia to build a similar argument. What Grossman (1992) argued is that Freud's theorizing suggests just such an underlying hierarchical model which involves discrete hierarchically defined systems with their own boundaries, where information at one level moves across boundaries as “representation” in another bounded system. At this point, it is hoped that the reader can observe the special and unique role that the FEP can play in such a description of the Freudian mind proposed by Grobbelaar (1989) and Grossman (1992). The FEP is useful here because it specifies just such a regulatory principle of the emergence of HRO within the nervous system, where hierarchically superordinate layers that emerge in the nervous system obtain self-organizing or Markov characteristics, and where message passing between layers is represented “upwards” as prediction error in progressively higher levels, and downwards as predictions (and the precisions of those predictions) to progressively lower levels. However, while the FEP provides a basis for formulating a HRO model of organization in the nervous system, it does not explain the specific operation of each of those levels and what they contribute toward the overall functioning of the system. What would be needed would be a description of the most relevant and proximal layers that most closely influence the level of interest which is that of psychoanalytic regulatory principles.

**Figure 3:**
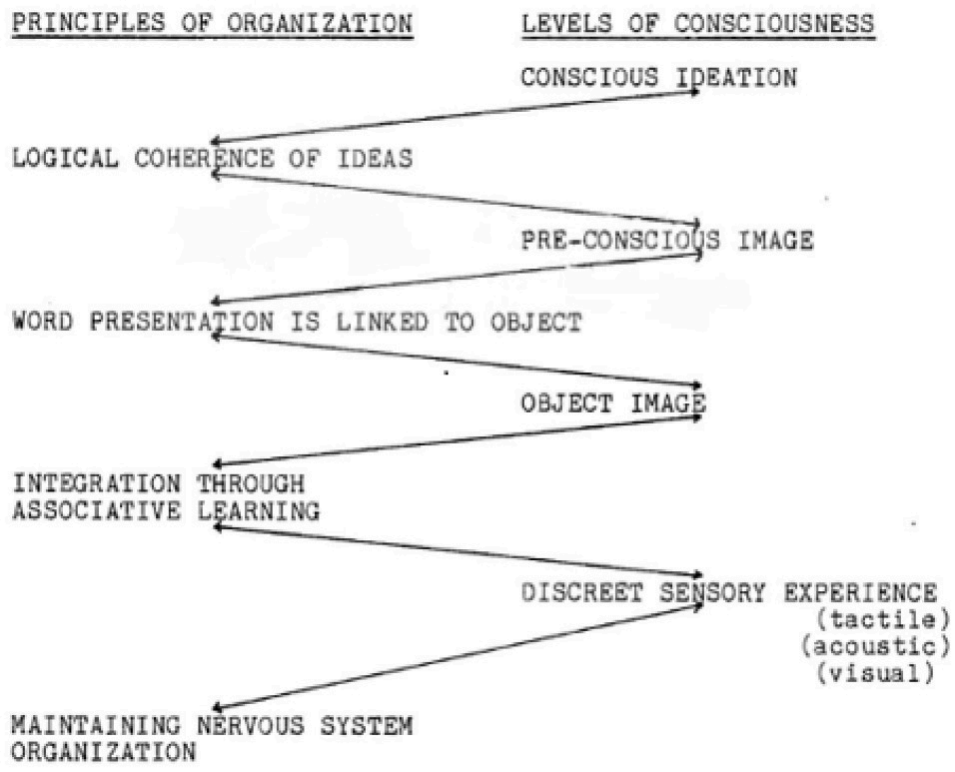
A recursive description of consciousness organized by language, after Grobbelaar (1989).

Grobbelaar does indeed go further in terms of offering such an example of a recursive description of the kind he calls for, represented diagrammatically in Figure 3 below.

In this diagram, Grobbelaar (1989) is presenting a recursive description of the organization of conscious experience which suggests that psychic material can only become conscious when it can be represented at successively higher levels of organization. Conversely, any psychic material which is not integrated into the (recursive and hierarchical) organization cannot become conscious. Following the dependence of consciousness on language in his recursive description here, we might say that whatever experience has not yet been integrated into the linguistic organization of our brains cannot become conscious, and that (to some extent) the ego could be defined as that part of our psyche which is organized by language.

However, Grobbelaar (1989) suggests a broader understanding of what is meant by representation:

“*The exclusivity of word-representation in allowing thoughts into consciousness should not be over-emphasized. Language should be seen as one of the most important organizing principles of experience, which acts as an entrance for experience to enter into consciousness. Its importance is linked partly to its inherent functional qualities but far more important is its quality of being used as an organizing principle. The important concept here is that experience has to be organized to have psychological meaning. It is obvious that language does not have a monopoly on this function. In the perception of music it is one's ability to perceive the rhythm and melodious organization which seems to be (a) independent of language (b) improved with repeated exposure (c) dependent on a different symbolic notation. The perception of visual pattern seems also to be dependent on an organizing principle other than language. It does, however, rely on language to an unknown extent where the visual pattern is an object which is also represented in our language … all these principles act as determinants of pattern discrimination. Without discriminating the pattern … there can be no awareness of the object.” (pp. 148–149)*

A last comment is made here about the lowest level of the recursive description found in Figure 3 above. It is important to note that this level of ordering refers to the maintenance of organization of the nervous system. In his thesis, Grobbelaar (1989) argued that this organizing tendency of language was itself recursively constituted from the tendency toward autopoiesis, or self-making in the organism. In this way he hoped to demonstrate how the psychological ordering of conscious experience was itself founded upon the autopoietic ordering of the organism. By these means, he sought to describe the ego as self-organizing. Though Grobbelaar did not have access to Friston's (2010a) FEP, there is nonetheless good consilience between his view and a formulation of the recursive ordering of information within hierarchical layers such as that found in the FEP.

This last point is also important to understanding the powerful role that interoceptive influence plays on the activity of the mind, throughout the feedback loops described here. It assumed here that interoceptive information must enter the hierarchical organization of the brain at a relatively lower level of organization than that described by Grobbelaar (1989) above. However, a central proposition of psychoanalysis is that the homeostatic requirements of the body in the form of interoceptive information are a fundamental driver of affect and motivation. Due to the genetic endowment of the brain, as well as the primary place of interoceptive information in the early life of the organism (Hobson et al., 2014)—especially *in utero*—and the narrow parameters within which internal organs usually remain, the precisions associated with interoceptive information are very high. Therefore, a large proportion of the surprise present in the nervous system emerges from interoceptive input, particularly when activating prototype emotions, and so, when high enough, can progress through successive feedback loops up every layer structure that reflects the organizational hierarchy described here, so that even a conscious stream of logically ordered thoughts can be constrained by nagging perceptions of hunger and thoughts about what to do about it. However, it is acknowledged that this important topic of the role of interoceptive input in HRO and the FEP requires additional attention beyond that given here.

Grobbelaar's (1989) work has identified two subordinate levels or organization that are critical to conscious experience. He has chosen the organization of entry to preconsciousness as the level of interest, and generated a recursive description of the dependence of conscious organization on that of language (or patterned representation more broadly), though he also signaled the importance of affect without developing it much further in his text. The organization of affect as a foundational principle for consciousness is not explored in this article, though the reader is referred to a paper by Hopkins (2016), entitled “Free energy and virtual reality in neuroscience and neuropsychoanalysis: a complexity theory of dreaming and mental disorder,” where he demonstrates how the interaction between mental states organized by conflicting emotions—attempting to minimize their respective free energy—underlie a process of conscious experience that is strongly consilient with that described by psychoanalytic theory.

The preceding sections have hopefully lain the groundwork for a key conclusion expressed here, which is that the ego must be understood as self-organizing (Grobbelaar, 1989), and that the specific nature of that self-organizing process is itself emergent from the FEP (Connolly, 2016). The ego is understood here as an associative structure occupying the higher levels of organization of the generative model, that comes to influence lower levels of the hierarchy. As such, it develops Markov characteristics that mean it (the ego) must be viewed as effectively self-organizing—and potentially self-evidencing as described by Hohwy (2016). Psychoanalysis is proposed here as being essentially that science of the self-organization of the ego that describes its relative inertia and resistance to change, while also describing the unique principles of organization that operate at this level.

## Conclusion

The aim of Grobbelaar's (1989) argument was to challenge the notion of a top down ordering process of organizing consciousness (such as the preconscious gate in Freud, 1900/1991), that could not be shown to emerge from a bottom up process, and to call for a reformulation of Freudian concepts that makes use of systemic principles such as HRO. In turn, the thrust of the present paper is to show how such a recursive description is precisely what is needed to correctly recognize the influence of biological self-organization (in the form of the FEP) on processes related to conscious experience that are of central interest to psychoanalysis. Equally, the paper has also tried to demonstrate the limitations of the FEP in fully explaining higher levels of organization within the person, and that the self-organizing nature of the ego demands distinct models, which are what psychoanalytic concepts can be understood as offering. It is hoped that the paper offers a compelling argument in this regard. Future work should re-examine the key theoretical constructs of psychoanalysis in order to offer a recursive description of their dependence on lower levels of organization in the brain, within the constraints of Friston's FEP.

## Author contributions

PC provided the key theoretical ideas and wrote the paper, and is responsible for submission. The paper would be the first related publication of a Ph.D. thesis completed in 2016. VvD was supervisor of the Ph.D. thesis.

### Conflict of interest statement

The authors declare that the research was conducted in the absence of any commercial or financial relationships that could be construed as a potential conflict of interest.

## Footnotes

^1^My thanks to Dr Karl Friston for his helpful remarks in clarifying ergodicity, Markov blankets and free energy.

^2^My thanks to reviewer Professor Jim Hopkins for helping with the development of this reading of the relationship between the FEP and NS, by pressing my thoughts of the role of HRO and the FEP in this direction.
